# Validity of a Non-Proprietary Algorithm for Identifying Lying Down Using Raw Data from Thigh-Worn Triaxial Accelerometers

**DOI:** 10.3390/s21030904

**Published:** 2021-01-29

**Authors:** Pasan Hettiarachchi, Katarina Aili, Andreas Holtermann, Emmanuel Stamatakis, Magnus Svartengren, Peter Palm

**Affiliations:** 1 Department of Medical Sciences, Occupational and Environmental Medicine, Uppsala University, 751 85 Uppsala, Sweden; magnus.svartengren@medsci.uu.se; 2 Spenshult Research and Development Center, 302 74 Halmstad, Sweden; katarina.aili@hh.se; 3 School of Health and Welfare, Halmstad University, 301 18 Halmstad, Sweden; 4 National Research Centre for the Working Environment, 2100 Copenhagen, Denmark; aho@nfa.dk; 5 Department of Sport Science and Clinical Biomechanics, University of Southern Denmark, 5230 Odense, Denmark; 6 Charles Perkins Centre, School of Health Sciences, Faculty of Medicine and Health, University of Sydney, Sydney, NSW 2006, Australia; emmanuel.stamatakis@sydney.edu.au

**Keywords:** physical behaviour, bedtime, sedentary behaviour, physical activity, ProPASS, accuracy, daily activity, objective measurement, posture

## Abstract

Body postural allocation during daily life is important for health, and can be assessed with thigh-worn accelerometers. An algorithm based on sedentary bouts from the proprietary ActivePAL software can detect lying down from a single thigh-worn accelerometer using rotations of the thigh. However, it is not usable across brands of accelerometers. This algorithm has the potential to be refined. *Aim*: To refine and assess the validity of an algorithm to detect lying down from raw data of thigh-worn accelerometers. Axivity-AX3 accelerometers were placed on the thigh and upper back (reference) on adults in a development dataset (n = 50) and a validation dataset (n = 47) for 7 days. Sedentary time from the open Acti4-algorithm was used as input to the algorithm. In addition to the thigh-rotation criterion in the existing algorithm, two criteria based on standard deviation of acceleration and a time duration criterion of sedentary bouts were added. The mean difference (95% agreement-limits) between the total identified lying time/day, between the refined algorithm and the reference was +2.9 (−135,141) min in the development dataset and +6.5 (−145,159) min in the validation dataset. The refined algorithm can be used to estimate lying time in studies using different accelerometer brands.

## 1. Introduction

The time spent in various body postures and physical activities are important for health [1]. Assessment of daily postures and physical activities are therefore of importance for health-related surveillance monitoring, risk assessments, advice and interventions. Traditionally, hip and wrist mounted accelerometers have been used to estimate physical activity intensity and energy expenditure [2]. However neither hip- or wrist worn accelerometers can accurately detect body postures during daily living [3]. Thigh worn accelerometers are well-suited for both detecting types of physical activity (e.g., walking, cycling, stair climbing and running) and body postures [4] and are frequently used to measure postures, sedentary behaviour and body movements continuously 24/7 [5,6]. In research about sedentary behaviour, it is important to differentiate between sedentary time and sleep. This is often done by using a bedtime or sleep diary [7]. However, this is burdensome to participants, and diary reports are subject to misreporting and compromised validity. Automatic identification of bedtime based on information about when participants have been lying down could be a feasible and viable option.

Algorithms for detection of postures and activities from thigh worn accelerometers use information from the accelerometer to calculate movements of the thigh and the angle of the thigh towards the line of gravity [4]. In principle, in situations when the movements are small and the thigh is horizontal, the participants are assumed to be sedentary. However as the thigh is horizontal, both when the subject is sitting or lying, differentiation between sitting and lying postures with only a thigh worn accelerometer is not straightforward. A way to come around this issue is to apply a second accelerometer on the back of the participant, being able to precisely differentiating between sitting and lying down from the estimated inclination of the back [8,9]. Using an additional accelerometer is disadvantageous as it brings extra burden on the participant and imply extra administrative costs.

Lyden et al. have presented an algorithm that instead uses information about rotation of the thigh to detect lying down episodes from a single thigh-worn accelerometer [10]. The idea behind this algorithm is that participants often roll over to the side while lying down. The rotation detection of this algorithm uses raw accelerometer data, but also relies on the detection of sedentary bouts from the proprietary ActivePAL algorithm. Any sedentary bout with rotations is therefore classified as a lying down bout. From here on this method will be referred to as the Lyden method. The Lyden method accurately identified 96% of the lying time, when compared to diary based information about nightly bedtime among 14 adult office employees [10]. Short lying episodes also might have occurred during daytime (thus not captured by the diary). The Lyden method has therefore also been compared with estimation of lying episodes in a dataset with 35 participants during 24 h in which the lying episodes were defined from a back accelerometer [11]. For most participants the Lyden method performed well. However, sitting with crossed legs can in theory be identified as lying (due to detected by the algorithm as thigh rotation), and might be one reason for overestimations in some cases. Underestimation may in theory happen, if a person is lying on his/her back without turning during lying down [11]. Thus, there is a potential to increase the accuracy of the Lyden method.

The Prospective Physical Activity, Sitting and Sleep consortium (ProPASS) aims to pool and harmonize studies of thigh-worn accelerometer data [5]. A prerequisite for fulfilling the goals of this consortium is the use of non-proprietary algorithms that relies on raw accelerometric data. Since the Lyden method is based on the proprietary ActivPAL software, there is also a necessity to test and validate it against the sedentary bout classification of an open algorithm such as Acti4, which works on raw-data from any accelerometer brand.

The aim was to integrate, and refine the method to identify lying down time, suggested by Lyden et al., with the open Acti4 algorithm and to validate this method in a dataset with additional back-worn accelerometer as the reference.

## 2. Materials and Methods

Participants to this study were recruited from the ongoing Halland County Knee Osteoarthritis (HALLOA) cohort longitudinal study. The inclusion criteria for HALLOA was (1) be 30–65 years old; (2) have knee pain; (3) no history of anterior cruciate ligament (ACL) injury; (4) no inflammatory rheumatic disease; (5) not have previously confirmed knee osteoarthritis. In addition, they should be in work to enter the present study. The population consisted of people who work day-shifts, night-shifts and also 24-h shifts. The 50 first participants, with valid accelerometer data, that joined the study were used in a development dataset. Then, the next 47 participants, with valid accelerometer data, from the same study were used in a validation dataset (Table 1). The criteria for valid accelerometer data are described in Section 2.3 data processing. The participants reported their work time and the time they went to bed and woke up in a diary each day. In a survey, the participants reported scorings from the knee injury and osteoarthritis score (KOOS) subscales “Pain”, activities of daily life “ADL”. A scoring (0–100) for each subscale was generated where a lower score indicate worse pain and worse physical functioning [12,13]. A research assistant measured the participants’ height and weight at the study centre. The data was collected between November 2018 and December 2019. The study was conducted in accordance with the Declaration of Helsinki, and the protocol was approved by the Ethics Committee in Lund, Sweden (Dnr 2018/602).

The development and validation dataset were found be comparable regarding distributions of gender, age, BMI, height, KOOS-Pain and KOOS-ADL using a non-parametric Mann Whitney U test (with significance values 0.98, 0.58, 0.32 and 0.86 respectively).

### 2.1. Instrumentation

All participants were equipped with two tri-axial accelerometers (Axivity AX3, Axivity Ltd., Newcastle, UK) by a research assistant at the study centre. The accelerometers were positioned at (1) the upper back (axial, on C7-Th1 level); (2) the right thigh (anterior, midway between the iliac crest and patella. The accelerometers were attached according to a standardized protocol for coherence of the three axis positions, and to get reference values for standing position using medical-grade self-adhesive tapes.

The participants were asked to wear the accelerometers during 7 days and nights. They were instructed to wear the accelerometers at all time (including bath) and only remove them if they experienced itching from the tape or any other discomfort. If removal of accelerometers occurred, or if the accelerometers fell off, they were instructed to note the time and contact the research assistant for further instructions.

### 2.2. Data Processing

The two accelerometers were time synchronized with a custom made Matlab algorithm. The algorithm synchronized the accelerometer signals by conducting a robust-fit linear regression of the time shift found by the cross-correlation of the two accelerometer signals under free living conditions. Before further analysis, accelerometer signals were calibrated automatically according to the method presented by Van Hees et al. [14]. In order to adjust for minor deviations in the back accelerometer placements, and shapes of the participants back, the raw tri-axial accelerometer data was corrected using a reference position when the participants were told to stand upright for 10 s. Similar correction for the thigh accelerations was performed daily, by identifying walking episodes during free living condition. In addition to non-wear periods noted down by the participant, an automatic non-wear detection built-in to the Acti4 algorithm was used during data processing. Both user-defined and auto detected periods of non-wear were excluded from the analysis.

Valid accelerometer data was defined as cases with both successful time synchronization and placement correction.

### 2.3. The Reference Method

**Figure 1:**
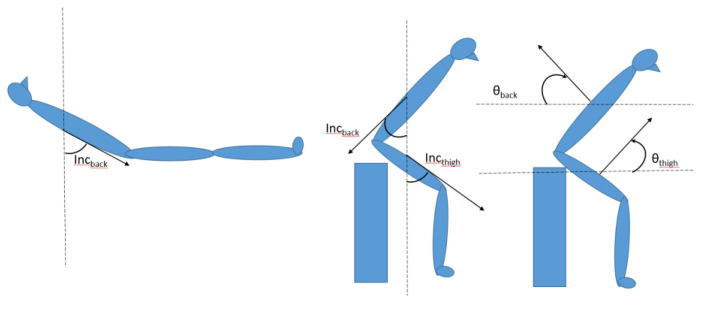
Definitions of the angles used in the equations.

As reference, all lying bouts were detected with the use of both the thigh and back accelerometer. The sedentary bouts of sitting and lying were first detected by the validated non-proprietary Acti4 algorithm for the thigh-worn accelerometer [4,15,16]. The primary criteria for differentiating of lying from sitting, from back accelerometer was an inclination angle of the back towards the line of gravity (*INC_back_*) above 45 degrees (Figure 1). The resultant lying bouts was then further filtered to remove possible cases of false positives caused by leaning forward while being in a sitting posture (Figure 1). This was done by first identifying lying on stomach episodes by using the sign of the angle of the sagittal axis coming out of the back (*θ_back_*), towards the horizontal plane. Positive angles of *θ_back_* indicated stomach lying or leaning forward. Then if the angle between the sagittal axis coming out of the front of the thigh and the horizontal plane (*θ_thigh_*), were more than 45 degrees during these stomach lying episodes, these were reclassified to sitting as follows:

(1) $$IF\;\theta_{back}>0\;AND\;\theta_{thigh\;}>45^{{}^{\circ}}\;THEN\to SIT$$

In order to filter out false positive lying episodes due to when the person was leaning sideways the following equation was used.

(2) $$IF\;ABS\left(INC_{back}-\theta_{back}\right)<10\;AND\;INC_{back}<65^{{}^{\circ}}AND\;\theta_{back\;}<65^{{}^{\circ}}\;THEN\to SIT$$

### 2.4. Development of the Algorithms

Two algorithms (A and B) were developed based on the sedentary classifications from the Acti4 algorithm. Algorithm A is a direct implementation of the lying detection according to the method described by Lyden et al. [10]. Algorithm B is a refined version of algorithm A. It was refined and optimized using the development dataset of the material described above.

**Figure 2:**
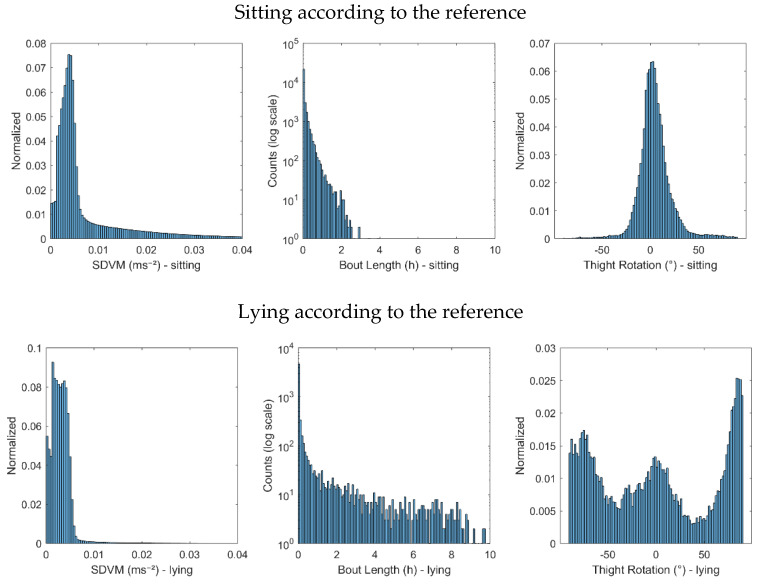
The distributions of parameters SDVM, time duration of sedentary bouts (bout lengths) and thigh rotation angle, during sitting and lying down, according to the reference method in the development dataset.

**Figure 3:**
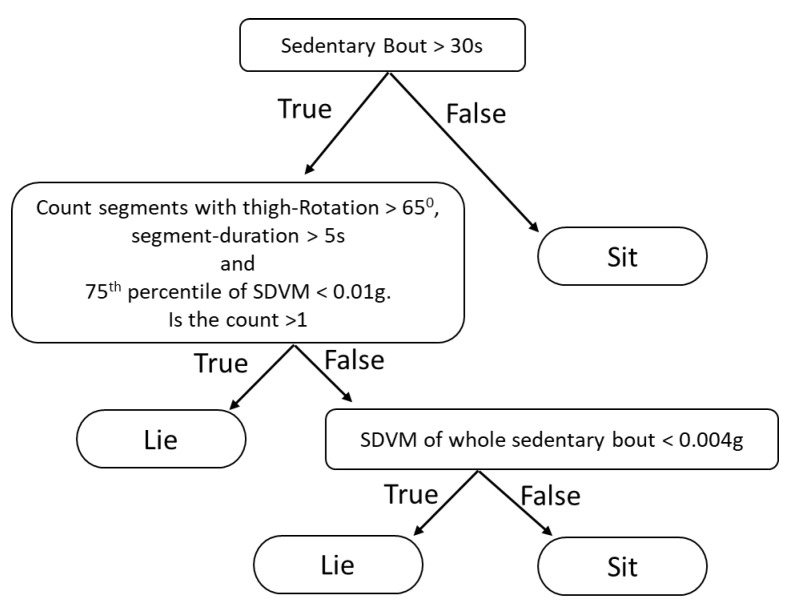
Decision tree of Algorithm B.

It was hypothesised that sitting with crossed legs may lead to false positive lying detection [11]. We hypothesised that when one is sitting with crossed legs, the thigh may move slightly more than when lying down, since the leg then is stabilized by the surface of the bed. We therefore evaluated the standard deviation of the vector magnitude of a 5 Hz low pass filtered acceleration (SDVM) during sitting bouts and lying bouts according to the reference method The right tail of the SDVM distributions differed between sitting and lying bouts (Figure 2). Therefore, an upper 75th percentile SDVM limit (SDVM_1) was included in Algorithm B (Figure 3).

Since the algorithm will not identify lying if the participants not rotates the thigh in the bed [11], we hypothesised that in these lying bouts the thigh is more likely to be still than during sitting because the thigh is supported by the bed. Therefore, a very low upper threshold of SDVM could potentially also be used to detect some of these situations. Therefore, the threshold SDVM_2 was included in Algorithm B to limit false negative cases.

We also hypothesised that true lying episodes are longer than such false positive cases. The distributions of the bout lengths indicated that sitting bouts were normally considerably shorter lying bouts (Figure 2). Therefore, a lower time duration limit (MinLieT) was included in Algorithm B.

During the development of the algorithm, we also found situations where the rotation angle crossed the threshold, but then very shortly after the crossing, went back below the threshold again. Therefore, momentary crossings with less than 5 s duration were not considered in Algorithm B.

Preliminary values for the included thresholds for SDVM_1, MinLieT and SDVM_2 were chosen based on the information from the distributions. Then a simple optimization procedure (by changing only a single threshold at a time) of the three thresholds was performed to achieve the maximum sensitivity plus specificity (see 2.6 statistics). The resultant thresholds were SDVM_1 = 0.01 g, MinLieT = 20 min, and SDVM_2 = 0.004 g respectively. In addition, by both investigating the distributions of the thigh rotation angles (Figure 2) and using the same optimization procedure, the thigh rotational angle threshold was also found to be 65 degrees as suggested by Lyden et al. The decision tree of Algorithm B is described in detail in Figure 3.

### 2.5. Validation of the Algorithms

Both Algorithm A and B were validated by applying those algorithms in the validation dataset and comparing the results from them with the reference method. The comparison was carried out using the sensitivity, specificity and a Bland-Altman analysis as described in 2.6 Statistics.

### 2.6. Statistics

The sensitivity and specificity as well as accuracy were calculated by comparing the classifications of lying down, between the reference method and both Algorithm A and B, for each second (Equations (3)–(5)). Since lying was fairly prevalent in the both datasets (36% and 35% of the total measured time respectively) in addition to sensitivity and specificity, accuracy was also deemed to be a valid performance index of the algorithms:

(3) $$Sensitivity=\frac{TP}{TP+FN}$$

(4) $$Specificity=\frac{TN}{TN+FP}$$

(5) $$Accuracy=\frac{TP+TN}{TP+TN+FP+FN}$$

TP = number of true lying-down seconds with respect to reference;

FP = number of false lying-down seconds with respect to reference;

TN = number of true non-lying-down seconds with respect to reference;

FN = number of false non-lying-down seconds with respect to reference.

The difference in lying down time duration, between the Algorithm A and the reference as well as Algorithm B and reference were calculated for each day. The mean differences in lying down time duration and standard deviation (SD) over all days were calculated.

For each participant, the averaged lying down time per day was also calculated by dividing the total lying down time by the each participants measured time (days). Then the mean values and SD for those differences were also calculated.

The 95% limits of agreement (LOA) was calculated by taking the mean differences ±1.96 times the standard deviation of the difference [17]. Only days with >8h wear periods from both back and thigh accelerometer were used in the analysis.

In order to investigate if the participants’ height, BMI, KOOS-pain or KOOS-ADL could influence the accuracy of the algorithm, correlations between the differences in detected lying time between Algorithm B, and the reference per subject and day were performed with Pearson correlation. IBM SPSS Statistics for Windows, version 21 (IBM Corp., Armonk, NY, USA) was used.

Manual inspections were performed to identify potential patterns in the errors. The 5% of the days in which lying down time differed the most, for Algorithm B in the validation dataset were inspected.

## 3. Results

In total there were 407 and 339 valid measured days in the development dataset and test dataset respectively. Averaged measured days per participant was 7.7 (SD ± 1.2) in the development dataset and 7.2 (SD ± 2.8) in the validation dataset.

Table 2 shows that the sensitivity to detect lying with Algorithm B was lower compared to Algorithm A, but the specificity was higher. There were no differences in accuracy between the two algorithms (Table 2).

The mean lying down times across all measured days was in the development dataset 7.7 (SD ± 3.1) hours and 7.7 (SD ± 3.1) hours in the validation dataset according to the reference.

**Figure 4:**
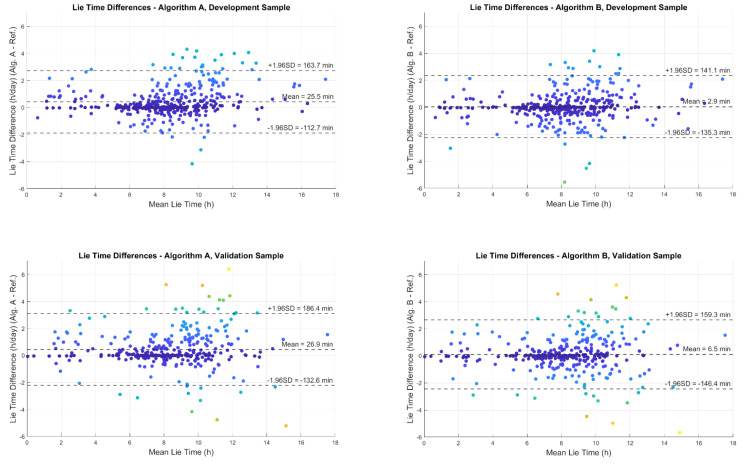
Bland Altman plots showing the difference in estimation of lying down time per day between the reference method and the two algorithms A and B. Each dot in the plots represent a measured day. The mean value of lying time by the reference method and each algorithm is given in *x*-axis. The difference between lying time detected by each algorithm and the reference method is given in *y*-axis.

Both Algorithm A and B overestimated the time of lying down compared to the reference method in both the development and validation datasets. The mean lying down time difference per day for Algorithm A compared to the reference was +25.5 min (SD ± 71), in the development dataset and +26.9 min (SD ± 81) in the validation dataset. The mean lying down time difference per day for Algorithm B compared to the reference method was +2.9 min (SD ± 70) in the development dataset and +6.5 min (SD ± 79) in the validation dataset. The time differences (calculated daily) and 95% limits of agreements are shown in Figure 4.

**Figure 5:**
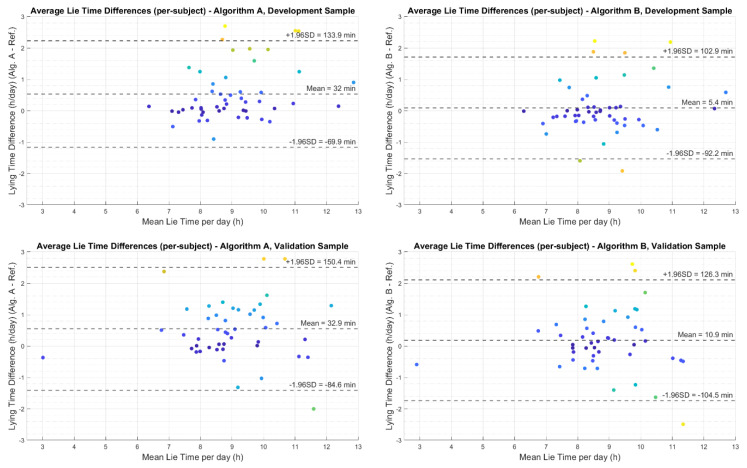
Bland Altman plots showing the difference in estimation of average lying down time per participant between the reference method and the two algorithms A and B. Each dot in the plots represent a participant. The mean value of lying time by the reference method and each algorithm is given in *x*-axes. The difference between lying time detected by each algorithm and the reference method is given in *y*-axes.

The mean lying down time difference per participant (calculated by first finding average lying down time of each participant and then taking the mean) for Algorithm A compared to the reference was +32 min (SD ± 51) in the development dataset and +32.9 min (SD ± 51) in the validation dataset. The mean lying down time difference per participant for Algorithm B compared to the reference method was +5.4 min (SD ± 49) in the development dataset and +10.9 min (SD ± 58) in the validation dataset. The time differences per participant and 95% limits of agreements are shown in Figure 5.

There were no correlations between the difference in detected time by Algorithm B and the reference method and the participants height (r=−0.25), BMI (r=−0.07), KOOS-pain (r=−0.01), or KOOS-ADL (r=0.02).

Out of the 18 days, that corresponded to the days with the 5% highest difference between lying down time according to Algorithm B and the reference, 13 days were derived from unique participants. In 17 of these days the major differences in classifications occurred in daytime during leisure, defined from the participants work diary.

## 4. Discussion

In this study, we developed two non-proprietary algorithms (A and B) aimed at detecting lying down time from raw tri-axial accelerometric data of a single thigh worn accelerometer. Algorithm A was a direct implementation of an earlier presented method by Lyden et al. The difference between Algorithm A and the Lyden method is that the Lyden method is based on definitions of sedentary bouts from the proprietary ActivePAL software [10] and Algorithm A is based on the validated and open Acti4 algorithm [4,15,16]. Algorithm A was then further refined into Algorithm B to handle some known issues with misclassifications. The performance of both A and B algorithms to identify lying down time was compared against a reference method including both thigh and back accelerometers to identify lying down time during free living conditions.

Algorithm A overestimated lying down time with 27 min per day compared to the reference in free living conditions (which corresponded to 6% of the average lying time). The Lyden method has been validated before, using a similar, but not identical, reference method, finding it to underestimate lying down time by 25 min per day in free living conditions [11]. These differences might be due to differences in the reference methods used. In the present study we introduced a correction in our reference that reclassified lying as sitting when the person was considered to sit leaning forward or sideways. This was, to our knowledge, not used in the earlier validations study [8]. This means that the reference we used would be more restrictive in identifying lying down time, which might explain the differences between the estimations.

Another difference is that the lying down detection is based on sedentary bouts detected by the proprietary ActivePAL software in the Lyden method and the Acti4 algorithm in our developed algorithms. Because of the proprietary nature of ActivPAL software, a comparison of these methods at the algorithm-level is not possible. Both the ActiPAL software and Acti4 algorithm are shown to be robust and valid methods to detect sedentary bouts [4,15,16,18,19]. However, if there are different level of filtration of sporadic activities within a given sedentary bout, this will affect how much of that period being classified as lying. Therefore, the full non-proprietary version of original Lyden algorithm implemented in this work could be somewhat different to the work described by Lyden et al.

The refined Algorithm B overestimated lying down time with +6.5 min per day compared with the reference, corresponding to 1.4% of the average lying time. Over 24 h this overestimation correspond to negligible 0.4% of the day. The precision was somewhat higher than observed for the Lyden method before [11].

For Algorithm B, 5% of the observed days were days where the lying estimation differed more than 173 min from the reference, corresponding to 12% of the time per day. When we manually inspected these 5% worst classified days we found that for almost all of these days the misclassifications had occurred during leisure within daytime. One probable explanation is that the misclassification occurred when the participants were half lying down (positioned between horizontal lying and sitting). This would then indicate an ambiguity in the definitions of is sitting and lying rather than a limitation in the lying down algorithm.

In very large dataset, errors may be of minor importance as long as the error is random and not biased by any systematic error due to personal characteristics or health related issues of interest. Neither, height, BMI, knee pain nor reduced ability to perform activities in daily life due to knee pain, affected the estimations of lying time in the present material. This indicates that there are no systematic errors due to these health related variables.

The studied population represented a variation in occupations and work-hours–including people who work day-shift, night-shift and also 24-h shift. It should be noted that if the algorithms should be used on other populations (ex. children) validity of the algorithms needs to be re-evaluated.

One general limitation with algorithms to detect lying based on information from rotations of thigh worn accelerometer is that if the person never rotates their thigh during lying period the algorithms fail to identify these situations. In Algorithm B we therefore introduced a criteria that if someone has been sedentary, with none or very small, movements that sedentary bout should be identified as lying. However, the acceleration threshold that was introduced in the Algorithm B for this purpose is very low and might, in theory, be below the minimum acceleration that can be measured by some earlier version of common devices with 8-bit resolution or less.

The present study offers non-proprietary algorithms for any accelerometer device that can output raw tri- axial accelerometer data. We have used the Axivity AX3 in this validation study but there are no reasons to believe that the results would be different with any other devices with similar acceleration range and resolution. This has been a demand from researchers and specifically from the ProPASS consortium that need such a tool for processing pooled raw thigh accelerometer data from cohorts around the world. Both Algorithm A and the improved Algorithm B be can be used together with the Acti4 algorithm when raw accelerometer data from different brands shall be pooled and processed in a harmonized way. Acti4 has already been empirically evaluated to be suitable for that purpose [16].

Automatically identifying bedtime, without the use of a diary, would highly decrease the cost and burden with both collecting and processing accelerometer data in large observational studies. So far, algorithms that automatically identifies bedtime from thigh accelerometers has been based on evaluation of the length and time patterns of sedentary bouts over the day [20,21]. We believe that such bedtime algorithms can be improved if they instead rely on lying bouts generated from the presented algorithms rather than sedentary bouts. However, this need to be further investigated against valid data on bedtime.

## 5. Conclusions

In this paper two non-proprietary algorithms to identify lying down time from thigh worn accelerometers have been presented and validated. One of the algorithms is a direct implementation of the earlier presented method by Lyden et al., and the other is a refined version of the first.

The study confirms the earlier presented accuracy of the method by Lyden, but now in a much larger dataset with a better reference. Both algorithms accurately identified 94% of the lying time. The improved version decreased bias in lying time detection from 27 min/day to 6.5 min/day compared the direct implementation of the earlier presented method by Lyden et al.

The algorithms can be used to estimate lying time in studies with pooled raw data from thigh worn accelerometers of different accelerometer brands.

## Figures and Tables

**Table 1:** Description of the population in the development and validation dataset.

	Development Dataset n = 50, 34 Women			Validation Dataset n = 47, 33 Women		
	Mean (SD)	Min–Max	Median (Interquartile Range)	Mean (SD)	Min–Max	Median (Interquartile Range)
Age	52.6 (7.9)	30–62	55.0 (11.5)	52.7 (8.3)	32–67	54.0 (12.0)
BMI	26.8 (5.1)	18.8–44.9	25.4 (7.8)	26.1 (4.3)	19.6–37.2	24.6 (6.3)
Height	170 (8.6)	153–186	169 (12.2)	172 (8.2)	160–194	171.0 (11.3)
KOOS-Pain	74.6 (17.7)	19.4–100	75 (26.3)	71.3 (16.6)	38.9–100	69.4 (25.0)
KOOS-ADL	79.1 (16.7)	19.1–100	79(23.5)	80.4 (15.1)	39.7–100	85.3 (26.1)

**Table 2:** Sensitivity and specificity of Algorithm A and Algorithm B for detecting lying down in the development dataset (n = 50) and the validation dataset (n = 47).

		Development Dataset		Validation Dataset	
		Algorithm A	Algorithm B	Algorithm A	Algorithm B
Sensitivity	Mean	0.97	0.94	0.95	0.94
	SD	0.03	0.05	0.05	0.06
	Min	0.81	0.71	0.75	0.75
	Max	1.00	1.00	1.00	1.00
Specificity	Mean	0.95	0.96	0.94	0.95
	SD	0.05	0.05	0.05	0.04
	Min	0.79	0.80	0.80	0.82
	Max	1.00	1.00	1.00	1.00
Accuracy	Mean	0.95	0.95	0.94	0.94
	SD	0.04	0.04	0.04	0.03
	Min	0.85	0.83	0.87	0.87
	Max	1.00	0.99	1.00	1.00

## Data Availability

The data that support the findings of this study are available from the corresponding authors, [P.H. or P.P.], upon reasonable request as long as it doesn’t compromise the privacy of research participants, or any Swedish or European Union regulations.

## Abbreviation

ACL	Anterior cruciate ligament
BMI	Body mass index
HALLOA	Halland county knee osteoarthritis cohort
*INC_back_*	Inclination angle of the back towards the line of gravity
KOOS-ADL	Knee injury and osteoarthritis score for activities of daily life
KOOS-Pain	Knee injury and osteoarthritis score for pain
LOA	Limits of agreement in Bland-Altman analysis
MinLieT	The threshold of minimum lying time
ProPASS	Prospective Physical Activity, Sitting and Sleep consortium
SDVM	Standard deviation of the vector magnitude of the 5 Hz low pass filtered acceleration
SDVM_1	Upper 75th percentile SDVM threshold 1
SDVM_2	Upper 75th percentile SDVM threshold 2
*θ_back_*	Angle between the sagittal axis coming out of the back and the horizontal plane
*θ_thigh_*	Angle between the sagittal axis coming out of the front of the thigh and the horizontal plane
